# Controlled‐Release Nanoparticles for the Treatment of Childhood Refractory Epilepsy in Latin America

**DOI:** 10.1155/bmri/3576367

**Published:** 2026-10-25

**Authors:** Tessy López-Goerne, Abel Santamaria, Antonela Gonzalez-Bondani, Rafael Valiente, Francisco Osuna-Orozco, Natalia Beltrán-Peña, Dulce Gurgua

**Affiliations:** ^1^ Department of Health Care Autonomous Metropolitan University—Xochimilco Mexico City Mexico uam.mx; ^2^ Department of Nanotechnology School of Science and Engineering Tulane University New Orleans Louisiana USA tulane.edu; ^3^ Faculty of Sciences National Autonomous University of Mexico Mexico City Mexico unam.mx

**Keywords:** controlled drug delivery, Latin America, nanomedicine, pediatric neurology, refractory epilepsy, sol-gel nanoparticles

## Abstract

Childhood refractory epilepsy (CRE) remains a major therapeutic challenge in Latin America, where limited access to specialized care, persistent treatment gaps, and high rates of pharmacoresistance contribute to poor clinical outcomes. This review examines the potential of sol‐gel–derived silica and titania nanoparticles as controlled‐release platforms for antiepileptic drug (AED) delivery, with particular emphasis on localized nanoreservoirs designed to improve drug retention at the epileptogenic focus while reducing systemic exposure. We summarize preclinical evidence for sol‐gel matrices loaded with conventional AEDs, including carbamazepine, valproic acid, phenytoin, and topiramate, highlighting their physicochemical properties, controlled‐release behavior, biocompatibility, and potential to enhance therapeutic efficacy. Particular attention is given to experimental strategies developed in murine temporal lobe epilepsy models, together with emerging approaches for targeted and minimally invasive brain delivery. We further discuss the translational pathway of these technologies, including regulatory considerations, Good Manufacturing Practice (GMP) requirements, pediatric clinical evaluation, and implementation challenges in resource‐constrained healthcare systems. By integrating materials science, experimental nanomedicine, and the socioeconomic realities of epilepsy care in Latin America, this review identifies the opportunities and remaining barriers for translating sol‐gel nanotechnology into clinically applicable therapies for CRE.

## 1. Introduction

Childhood refractory epilepsy (CRE), also called drug‐resistant epilepsy, is characterized by frequent, disabling seizures that persist despite adequate trials of two or more appropriate antiepileptic drugs (AEDs) and often limit a child′s normal neurodevelopment [1]. Globally, epilepsy affects about 4% of children, yet up to half of those cases become refractory, creating a disproportionate burden of morbidity and mortality in low‐ and middle‐income regions [2, 3]. In Latin America, prevalence estimates for pediatric epilepsy range from 3.5 to 11 per 1000 children, and 30%–50% of these patients develop treatment‐resistant forms. Mortality is consequently higher than in high‐income countries [4]. The challenges faced by children with refractory epilepsy (RE) are particularly pronounced in Latin America, where delayed diagnosis, shortages of pediatric neurologists, limited availability of epilepsy surgery, inconsistent access to antiepileptic medications, and high out‐of‐pocket healthcare expenditures contribute to one of the largest epilepsy treatment gaps worldwide. These regional characteristics make Latin America an especially relevant setting in which to evaluate therapeutic strategies capable of improving treatment effectiveness while reducing healthcare utilization and long‐term costs [5].

The socioeconomic toll is severe. Families must often purchase medicines out of pocket, whereas public health systems cover little beyond basic consultations and occasional hospitalizations. Children with uncontrolled seizures experience higher rates of cognitive impairment, social exclusion, and comorbid medical conditions, further straining limited educational and healthcare resources [6]. Addressing CRE therefore represents both a clinical priority and a public health imperative for Latin America and other resource‐constrained regions [7]. Nevertheless, AEDs suffer from low oral bioavailability, unpredictable brain penetration, and dose‐limiting adverse effects, ranging from hepatotoxicity and Stevens–Johnson syndrome to profound cognitive dulling [8, 9]. Even under optimal dosing, seizures persist in roughly 20% of patients with generalized epilepsies and 35% with focal epilepsies [10].

Surgical interventions such as vagus nerve stimulation (VNS) or corpus callosum callosotomy can reduce seizure frequency. However, they are invasive, costly, and accessible to only a fraction of children in low‐resource settings [11, 12]. Likewise, conventional AEDs often fail to achieve sustained therapeutic concentrations within the central nervous system (CNS) because of limited blood–brain barrier (BBB) penetration, rapid systemic distribution, and dose‐limiting adverse effects. Consequently, an ideal therapeutic strategy for CRE should not necessarily rely on new AEDs, but rather on improving how existing drugs are delivered to the epileptogenic tissue. Such an approach could prolong local drug exposure, reduce systemic toxicity, decrease dosing frequency, and ultimately improve treatment adherence.

Nanotechnology‐based drug delivery systems have emerged as a promising strategy to address these limitations by modifying the pharmacokinetic behavior of existing AEDs rather than their pharmacological mechanism of action. Engineered nanocarriers can protect drug molecules from premature degradation, facilitate transport across biological barriers, enable sustained drug release, and increase drug accumulation at the target site, thereby improving therapeutic efficacy while minimizing systemic exposure [13]. Among the different nanocarrier platforms investigated for CNS drug delivery, the sol‐gel process offers several advantages for pediatric applications [14]. Conducted at low temperature and pressure, sol‐gel chemistry produces highly pure, monodisperse silica or titania nanoparticles with tunable porosity and surface chemistry [15]. AEDs can be incorporated directly into the inorganic matrix during synthesis, forming nanoreservoirs capable of sustained and localized drug release while maintaining high drug dispersion and structural stability [16, 17].

The therapeutic potential of sol‐gel–derived nanoparticles lies in their ability to tailor drug release according to clinical needs. Particle size (1–100 nm), pore volume, and surface chemistry can be tuned by adjusting water‐to‐alkoxide ratios, gelation pH, solvent polarity, and catalyst choice, directly dictating drug‐release kinetics and brain‐tissue compatibility [18, 19].

Against this background, this review examines how controlled‐release sol‐gel nanocarriers may overcome some of the principal limitations of current epilepsy management by improving localized drug delivery, reducing systemic toxicity, and facilitating sustained therapeutic concentrations at the epileptogenic focus. We further discuss the clinical rationale, materials science, preclinical evidence, and translational challenges associated with these platforms, with particular emphasis on CRE in Latin America.

## 2. Clinical Background of CRE

### 2.1. Clinical Diagnosis and Classification of CRE

The diagnosis of CRE extends beyond confirming pharmacoresistance and requires accurate identification of the underlying epilepsy syndrome, seizure type, and etiology. This comprehensive evaluation is essential for selecting the most appropriate therapeutic strategy, including optimization of pharmacological treatment, epilepsy surgery, or emerging targeted drug‐delivery approaches.

Diagnosis typically involves a comprehensive clinical evaluation encompassing a detailed seizure history, family history, physical and neurological examination, and complementary tests such as electroencephalography (EEG) and neuroimaging (e.g., MRI and PET) [20, 21]. Together, these assessments allow clinicians to classify epilepsy syndromes, identify structural or metabolic abnormalities, and localize the epileptogenic focus when present, providing the basis for individualized treatment planning.

Once drug resistance has been established according to current International League Against Epilepsy (ILAE) criteria, early diagnostic characterization becomes particularly important in pediatric patients because recurrent seizures occur during critical stages of brain development. Persistent seizure activity has been associated with developmental delay, cognitive impairment, behavioral disorders, and reduced quality of life (Figure 1) [22]. In Latin America, where access to specialist care and advanced diagnostic tools is limited, the diagnosis of CRE is often delayed or incomplete, exacerbating its long‐term consequences [23].

**Figure 1:**
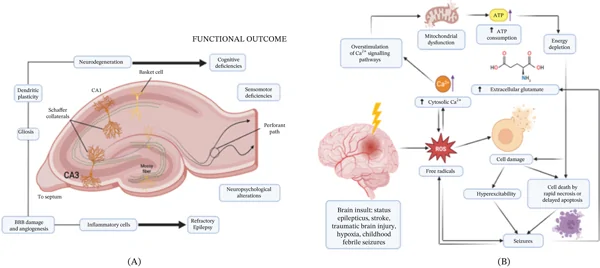
Refractory epilepsy. (A) Pathophysiology of CRE. (B) Status epilepticus mechanism and its involvement in CRE. Figure made in BioRender.com (accessed on October 25, 2025).

### 2.2. Pathophysiology of CRE

Understanding the pathophysiology of epilepsy, and particularly why some cases become drug‐resistant, is essential for the development of more effective therapeutic strategies. Two core processes must be distinguished: epileptogenesis (the transformation of a normal brain into one prone to spontaneous seizures) [24] and ictogenesis (the mechanisms that trigger and sustain individual seizures) [25].

In a healthy brain, neuronal communication is governed by a delicate balance between excitatory and inhibitory neurotransmission. Glutamate, the primary excitatory neurotransmitter, and gamma‐aminobutyric acid (GABA), the primary inhibitory neurotransmitter, maintain this balance [26]. In epilepsy, this equilibrium is disrupted, leading to excessive and synchronous neuronal firing [27], whereas, ictogenesis refers to the dynamic cellular and network‐level changes that lead to seizure onset. At the same time, epileptogenesis encompasses the long‐term molecular, structural, and functional changes—such as gliosis, synaptic reorganization, and neuroinflammation—that transform the brain into a chronically epileptic state (Figure 2) [28].

**Figure 2:**
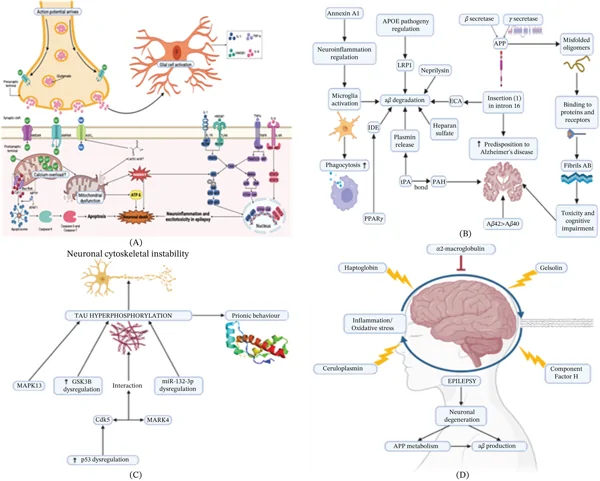
Pathophysiology of epilepsy and seizures. (A) Amyloid‐*β* (A*β*) protein degradation pathways associated with epilepsy pathogenesis and Alzheimer′s disease. (B) Self‐perpetuating molecular mechanisms involved in A*β* production and neuronal injury. (C) Representative endogenous and exogenous stimuli contributing to seizure initiation. (D) Neuronal damage and neurodegenerative processes associated with recurrent seizures. Figure 2 made in BioRender.com (accessed on October 25, 2025).

Because pharmacoresistance cannot be explained by a single biological mechanism, several complementary hypotheses have been proposed to account for the development of RE. These mechanisms involve alterations in drug transport, pharmacokinetics, molecular drug targets, genetic factors, disease severity, and pathological network remodeling, as summarized in Table 1.

**Table 1:** Hypotheses on refractory epilepsy.

	Description
Transporter [29]	Suggests that altered expression of drug transporters, particularly at the blood–brain barrier (BBB), reduces AED concentrations within the central nervous system. Overexpression of efflux transporters (e.g., P‐glycoprotein) may actively remove drugs from the brain, thereby limiting their therapeutic efficacy.
Pharmacokinetic [30]	Proposes that alterations in drug absorption, distribution, metabolism, or elimination reduce systemic and cerebral exposure to AEDs. Changes in transporter expression or metabolic activity in the intestine and liver may contribute to subtherapeutic drug concentrations.
Target [31]	Suggests that structural or functional alterations in molecular drug targets (e.g., ion channels or neurotransmitter receptors) reduce AED binding affinity or pharmacological responsiveness, thereby diminishing therapeutic efficacy.
Intrinsic severity [32]	Proposes that recurrent, severe seizures promote progressive neuronal injury, network remodeling, and maladaptive plasticity, ultimately reducing responsiveness to pharmacological treatment.
Gene variant [33]	Attributes pharmacoresistance to genetic polymorphisms that influence drug metabolism, transporter expression, or the sensitivity of molecular drug targets.
Neural network [34]	Emphasizes that chronic epilepsy induces structural and functional reorganization of neuronal circuits, resulting in pathological networks that become intrinsically less responsive to conventional pharmacological interventions.

Collectively, these hypotheses suggest that pharmacoresistance arises from multiple biological processes acting simultaneously. Consequently, therapeutic strategies solely aimed at increasing drug dosage may be insufficient. Alternative approaches capable of improving drug delivery to the epileptogenic focus, maintaining sustained local drug concentrations, or bypassing pharmacokinetic limitations have therefore attracted increasing attention, providing the rationale for nanoparticle‐based drug‐delivery systems discussed in later sections.

### 2.3. Epileptic Syndromes in Children

The ILAE classifies pediatric epileptic syndromes based on a combination of clinical features, EEG patterns, age of onset, and underlying etiology (Figure 3) [35].

**Figure 3:**
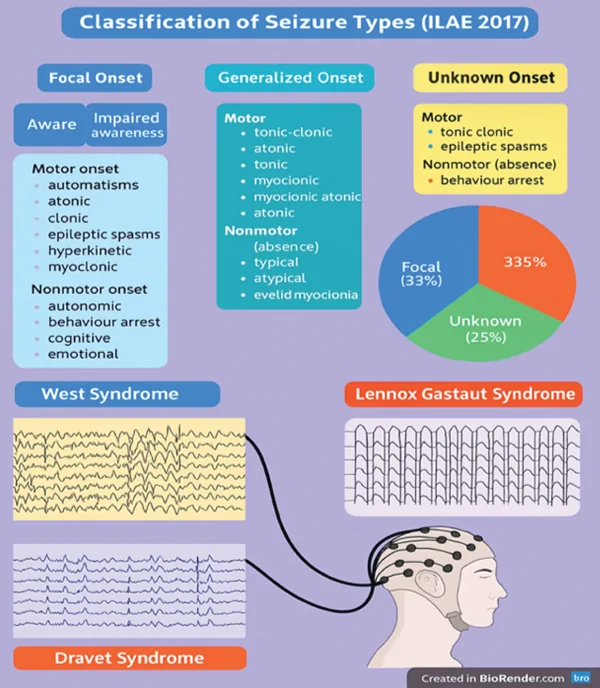
Classification of seizure types according to ILAE 2017 and etiologies incidence. Figure made in BioRender.com (accessed on October 25, 2025).

These syndromes span a broad clinical spectrum, ranging from benign, self‐limiting conditions to severe, treatment‐resistant epileptic encephalopathies associated with significant neurodevelopmental impairment. The primary syndromes are summarized in Table 2.

**Table 2:** Epileptic syndromes in children.

Syndrome	Age of onset	Main symptoms	EEG	Prognosis
Benign epilepsy with centrotemporal spikes [36]	3–13 years	Nocturnal focal seizures, facial paraesthesia	Centrotemporal spikes	Excellent, self‐resolving
Childhood absence epilepsy [37]	4–10 years	Brief absences, multiple per day	3‐Hz spike‐wave	Good with treatment
Benign myoclonic epilepsy of infancy [38]	4 months–2 years	Isolated myoclonia, no impairment	Normal or minimal alterations	Excellent
Benign neonatal seizures [39]	First days of life	Brief generalized seizures	Normal EEG between seizures	Excellent
Doose syndrome [40]	2–6 years	Myoclonia, atonic, and generalized seizures	Generalized spike‐waves	Variable
Self‐limited epilepsy with autonomic seizures [41]	1–14 years (peak 3–6)	Long focal seizures, autonomic symptoms (vomiting, etc.)	Intermittent occipital waves	Generally good
Childhood complex partial epilepsy [42]	< 10 years	Focal seizures with impaired awareness	Focal variable activity	Variable, treatable
West syndrome [43]	3–12 months	Spasms, psychomotor regression	Hypsarrhythmia	Severe, may progress
Lennox–Gastaut syndrome [44]	1–8 years	Multiple seizure types (tonic, atonic, atypical absences)	Slow spike − wave < 2.5 Hz	Very guarded
Dravet syndrome [45]	< 1 year	Prolonged febrile seizures, myoclonia, photosensitivity	Polymorphic, worsens progressively	Severe and resistant
Ohtahara syndrome (EIEE) [46]	Neonatal	Tonic seizures, severe impairment	Burst‐suppression pattern	Very severe

Among the self‐limited epilepsies, benign epilepsy with centrotemporal spikes (BECTS) is one of the most common and typically manifests between the ages of 3 and 13 years [47]. It presents with nocturnal focal seizures and is associated with an excellent long‐term prognosis. Childhood absence epilepsy is another well‐recognized self‐limited syndrome, characterized by brief, daily episodes of impaired awareness that generally respond well to pharmacologic treatment (10.1016/j.yebeh.2020.107342; 10.3390/pediatric13040078). Benign myoclonic epilepsy of infancy also falls within this category, typically resolving spontaneously after early childhood. It features isolated myoclonic jerks and usually exhibits minimal EEG abnormalities [48].

In contrast, several syndromes present with variable prognoses depending on individual response to treatment. Doose syndrome, or myoclonic–atonic epilepsy, commonly manifests between the ages of 2 and 6 years with a combination of myoclonic and atonic seizures [49]. The course of the disease can be unpredictable, with outcomes ranging from complete seizure control to severe cognitive decline.

Self‐limited epilepsy with autonomic seizures, often peaking between ages 3 and 6, presents with prolonged focal seizures accompanied by autonomic symptoms such as vomiting and pallor [50]. Although most cases resolve with age, the prolonged nature of seizures can complicate diagnosis and management. At the more severe end of the spectrum are syndromes associated with poor prognoses and substantial drug resistance. West syndrome typically presents between 3 and 12 months of age with epileptic spasms and psychomotor regression and is classically associated with a chaotic EEG pattern known as hypsarrhythmia [51]. Lennox–Gastaut syndrome (LGS) is a debilitating disorder that emerges between 1 and 8 years of age and is marked by multiple seizure types—including tonic, atonic, and atypical absence seizures—along with intellectual disability and a slow spike‐wave EEG pattern [52]. Dravet syndrome, a severe myoclonic epilepsy of infancy, begins in the first year of life with prolonged febrile seizures, progressing to multiple seizure types and developmental delay [53]. It is notoriously resistant to standard AEDs. Finally, Ohtahara syndrome, also known as early infantile epileptic encephalopathy, is a neonatal‐onset condition characterized by tonic seizures and a burst‐suppression EEG pattern. This syndrome is frequently linked to structural brain abnormalities and has one of the most severe prognoses among pediatric epilepsies [54].

Recognizing and correctly classifying these syndromes is essential, as each has distinct implications for prognosis, pharmacologic management, and potential surgical intervention. Early identification can guide the implementation of more targeted therapies and inform realistic expectations for developmental outcomes.

### 2.4. Current Treatment Limitations

The cornerstone of epilepsy management remains pharmacological treatment, with over 30 AEDs currently in clinical use. These medications act through diverse mechanisms, including modulation of voltage‐gated sodium and calcium channels, enhancement of GABAergic inhibitory neurotransmission, and suppression of glutamatergic excitatory neurotransmission [55]. By targeting various pathways involved in neuronal excitability, AEDs are aimed at stabilizing brain electrical activity and prevent seizure onset and propagation.

New drugs are still being developed, but they are not more effective than older ones, although they cause fewer adverse effects [56, 57]. It generates a need to find alternative treatments for epilepsy that can be accessible to all, especially in developing countries, where drug resistance is a significant problem due to the lack of resources to access current alternative treatments. Although RE is drug‐resistant, many treatments use AEDs as a complement. Among 29 studies conducted, it was observed that the use of drugs as a complement to treatment for refractory focal epilepsy was better for seizure control than in placebo groups. However, doses must be controlled to reduce the occurrence of unwanted side effects [58].

Despite their widespread use, AEDs are associated with several important limitations. Many of these compounds exhibit poor oral bioavailability due to extensive first‐pass metabolism, limiting the proportion of the active drug that reaches systemic circulation [59]. Even when absorbed efficiently, penetration of the BBB remains a significant hurdle, often resulting in subtherapeutic concentrations within the CNS (Figure 4). Additionally, individual variability in drug metabolism and the potential for complex drug–drug interactions make it difficult to achieve consistent therapeutic effects across different patients. These factors contribute to the narrow therapeutic windows of many AEDs, necessitating close monitoring to avoid toxicity while attempting to achieve seizure control [60].

**Figure 4:**
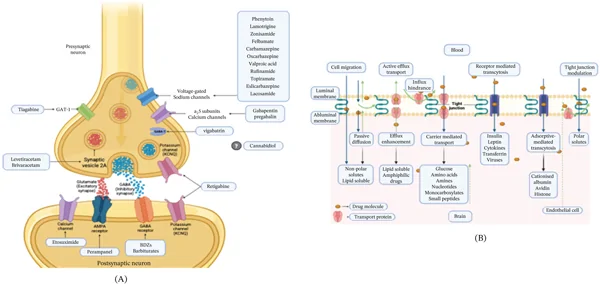
(A) Receptors and ionic channels in refractory epilepsy. (B) Mechanism of the BBB and the poor penetration. Figure made in BioRender.com (accessed on October 25, 2025).

Adverse effects are common and often dose‐limiting. Depending on the drug and dosage, patients may experience hepatotoxicity, sedation, cognitive slowing, dizziness, mood disturbances, or skin reactions such as Stevens–Johnson syndrome [61]. These side effects not only reduce quality of life but also contribute to poor adherence, especially in pediatric populations, where neurodevelopmental impacts are of particular concern. As outlined in Table 3, traditional AEDs such as valproic acid, carbamazepine (CBZ), phenytoin, and phenobarbital have been mainstays of treatment for decades [62]. However, their pharmacokinetic and safety profiles have remained essentially unchanged over time, and limited innovation in drug‐delivery systems has limited their clinical efficacy. In many patients with RE, prolonged treatment leads to diminished responsiveness, necessitating the use of multiple AEDs in combination [63]. This polypharmacy approach, although sometimes beneficial, often exacerbates adverse effects and further complicates disease management.

**Table 3:** Main antiepileptic drugs introduced to Europe in chronological order [64].

First generation	Second generation	Third generation
Bromide (1857), phenobarbital (1912), phenytoin (1960), primidone (1960), sulthiame (1960), carbamazepine (1965), valproate (1970)	Clobazam (1979), vigabatrin (1989), oxcarbazepine (1990), lamotrigine (1991), gabapentin (1994), felbamate (1994), topiramate (1995), tiagabine (1996), levetiracetam (2000), pregabalin (2005), zonisamide (2007), stiripentol (2007), rufinamide (2007)	Eslicarbazepine (2010), lacosamide (2010), retigabine/ezogabine (2011), perampanel (2012), everolimus (2017), brivaracetam (2018), cannabidiol (2019)

For patients with highly RE, particularly those with syndromes such as LGS or Dravet syndrome, surgical interventions may be considered when pharmacologic strategies fail [65]. Two notable options are VNS and corpus callosotomy (CC). VNS involves the implantation of a device that delivers intermittent electrical pulses to the vagus nerve, modulating neural activity to reduce seizure frequency and severity [66]. In contrast, CC is a more invasive neurosurgical procedure that involves severing the corpus callosum, the primary commissural pathway between the two cerebral hemispheres, to prevent the spread of epileptic discharges [67].

Both procedures have demonstrated moderate efficacy in reducing seizure burden. However, CC has been particularly effective in patients experiencing drop attacks, offering significant seizure reduction in selected cases [12]. However, these interventions are resource‐intensive, require specialized surgical expertise, and entail procedural risks. Their availability remains limited in low‐resource settings such as Latin America, where access to tertiary care centers and neurosurgical infrastructure is often restricted [68]. As a result, many children with RE in these regions remain without access to potentially beneficial surgical therapies, underscoring the urgent need for alternative strategies that are less invasive, more accessible, and better suited to pediatric use.

### 2.5. Epilepsy Metabolism

Metabolic disorders play a critical role in the pathogenesis of seizures, particularly within specific inherited conditions:

• Amino and organic acidopathies. The therapeutic approach to these epilepsies differs from the classic approach to the evaluation and management of epilepsy [69]. Early myoclonic encephalopathy and myoclonic seizures represent a classic epileptic syndrome and a seizure type, respectively, associated with inborn errors of metabolism, as can be seen in Figure 5 [70]. Neonatal hypoxic‐ischemic encephalopathy or the presence of structural abnormalities resulting in absence epilepsy with an underlying metabolic pathophysiology is suspected [71]. In addition, there are imaging findings suggestive of a metabolic disorder. Such as callosal dysgenesis in pyruvate dehydrogenase complex deficiency, intraventricular choroid plexus cysts in Zellweger syndrome, and enlarged Sylvian fissures in glutaric aciduria Type 1 [72–74]. Aminoacidopathies and organic acidopathies result from the accumulation of toxic intermediates or possibly from structural damage and usually present with myoclonic seizures and cognitive, behavioral, or motor disturbances. They may progress to epileptic encephalopathy [75, 76].

• Hyperhomocysteinemia. The spectrum of neurological dysfunction in homocysteine metabolism disorders is broad and includes epilepsy, encephalopathy, peripheral neuropathy, ataxia, microcephaly, and psychiatric disorders [77]. Pyridoxine, cyanocobalamin, folate, and a low‐methionine diet are used for cystathionine beta‐synthase deficiency, whereas high‐dose hydroxycobalamin is useful for methionine synthase deficiency [78–80]. Also, methylenetetrahydrofolate reductase deficiency, inherited in an autosomal recessive manner, can present in early childhood with status epilepticus, severe epileptic encephalopathy, and infantile spasms, and progress to LGS [81–85].

**Figure 5:**
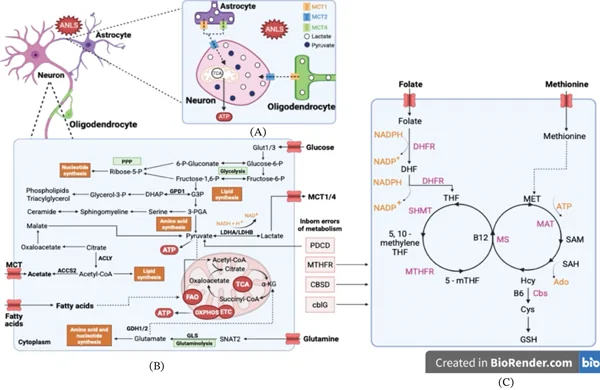
Epilepsy metabolism and inborn errors that can lead to drug resistance. (A) Epilepsy metabolism, including the PDC (pyruvate dehydrogenase complex) deficiency, is an inborn error that disrupts the normal production of energy. (B) Mechanism of the metabolism during a seizure. (C) Homocysteine metabolism, including folate and methionine cycle. Additionally, MTHFR (methylenetetrahydrofolate reductase), CBS (cystathionine beta‐synthase), and cblG (methionine synthase) inborn errors can affect these cycles and, in turn, lead to drug resistance.

Epileptic and early childhood developmental encephalopathy have been associated with these disorders. Methylmalonic acidemia is the most common cobalamin‐related disorder in cobalamin C deficiency; seizures may occur in infancy or early childhood, including status epilepticus and progressive encephalopathy [86, 87]. Prenatal and postnatal hydroxocobalamin supplementation is effective but may not reverse neurological damage [88]. When there are defects in serine synthesis, RE is sure to occur in the newborn, as well as in the postnatal stage [89]. Oral serine and glycine supplementation can improve outcomes; unfortunately, this treatment remains experimental [90, 91]. When there is insufficient sulfite oxidase, seizures are intractable, primarily myoclonic or tonic‐clonic, and begin in the first weeks of life and can progress to status epilepticus [87, 92]. Low total plasma homocysteine is associated with both, and hypouricemia due to a secondary xanthine dehydrogenase deficiency may indicate a molybdenum (Mo) cofactor deficiency [93]. Treatment has historically been symptomatic, but cyclic pyranopterin monophosphate is now available for Mo type A cofactor deficiency [94].

• Fatty acid oxidation disorders. Seizures can be a presenting sign of fatty acid oxidation disorders, a broad category of diseases that primarily affect the CNS and other organ systems with high energy demands. Metabolic decompensation can occur at any age and is triggered by stress, physical exertion, and exposure to cold, causing shivering and thermogenesis. Acute seizures resemble Reye′s syndrome, with cardiomyopathy, arrhythmia, rhabdomyolysis, and hypoketotic hypoglycemia [95]. Treatment consists of avoiding these conditions and consuming a high‐carbohydrate, low‐fat diet. Carnitine therapy is helpful in cases of a carnitine transporter defect [96].
• Glucose transporter one deficiency. Glucose transporter 1 (GLUT1) facilitates glucose transport across the BBB, and its limited distribution in the developing brain leads to metabolic encephalopathy [97].

CSF shows hypoglycorrhachia associated with normal plasma glucose and lactate levels. A wide range of phenotypes has been associated with this disorder, but 90% of affected children develop epilepsy (of various types, including absence, focal, generalized myoclonic, clonic, tonic, and nonconvulsive status epilepticus). Long‐term treatment with the ketogenic diet, which replaces glucose with ketone bodies as the primary source of biochemical energy, produces a rapid response, even in patients with refractory seizures. Compounds that inhibit GLUT1, such as phenobarbital, diazepam, valproate, methylxanthines (theophylline and caffeine), and alcohol, should be avoided [98].

## 3. Socioeconomic Challenges in Latin America

RE presents a significant public health burden in Latin America, where its management is complicated by a convergence of socioeconomic, infrastructural, and healthcare disparities. Although the prevalence of epilepsy in children ranges from 3.5 to 11 per 1000 across the region [99, 100], studies estimate that between 30% and 50% of these children have RE—forms of the disease that do not respond to standard antiepileptic treatments [101]. Despite its high prevalence, epilepsy remains widely underdiagnosed and undertreated in many Latin American countries, particularly among impoverished and rural populations.

Access to healthcare is one of the most critical barriers. In countries such as Mexico, Nicaragua, and Guatemala, significant gaps persist in the availability and distribution of neurologists, diagnostic tools like EEGs and MRIs, and antiepileptic medications. Although some urban areas may offer specialized care, these services are scarce or altogether absent in rural regions. For example, in Mexico, although infrastructure and medical specialists are more abundant compared with some neighboring countries, access to care is still primarily restricted to urban centers, leaving those in marginalized communities with little to no medical support. It is estimated that between 60% and 90% of people with epilepsy in Latin America do not receive appropriate treatment [102]. This treatment gap is further widened by systemic issues within national healthcare systems. Many countries in the region have struggled with stagnant public health funding, inadequate rural health coverage, and overburdened medical services. As a result, families often shoulder the financial burden of treatment themselves, purchasing out‐of‐pocket medications. This cost can be prohibitively high given the widespread poverty in the region. Public healthcare systems generally cover only basic consultations and emergency services. Surgeries for epilepsy are rare and usually performed at only a handful of tertiary hospitals, where resources are minimal. It is estimated that only 2–3 epilepsy surgeries are conducted annually in many Latin American countries, despite the large number of children who might benefit from such interventions [23].

In addition to structural limitations, social factors also play a role. Epilepsy in Latin America is frequently surrounded by stigma, misinformation, and cultural misconceptions, which can lead to delayed diagnosis or nonadherence to treatment [103, 104]. Traditional healers and naturopaths still serve as primary care providers in many rural communities, often because formal medical treatment is unaffordable. Although these informal systems can sometimes offer supportive care, they are not substitutes for evidence‐based medical treatment and may contribute to poor disease control and chronic disability (Figure 6).

**Figure 6:**
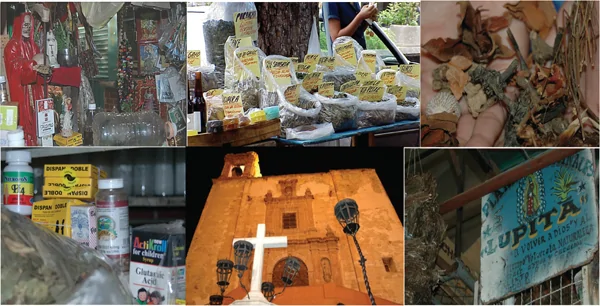
Traditional healing practices as a reflection of the treatment gap in chronic wound care. In many regions, particularly where access to specialized wound care is limited, patients may turn to traditional healers or other last‐resort therapeutic options. These practices illustrate the persistent treatment gap faced by individuals with chronic wounds and underscore the need for accessible, evidence‐based regenerative therapies capable of improving clinical outcomes across diverse healthcare settings.

Educational disparities further exacerbate the impact of childhood epilepsy. Children with poorly managed seizures often experience academic difficulties and social exclusion, limiting their long‐term opportunities. It is especially detrimental in regions where access to inclusive education and developmental support is already inadequate. In low‐income families, particularly those with multiple children and limited access to public services, children with epilepsy are at increased risk of neglect, undernutrition, and untreated comorbidities [105, 106]. Malnutrition and infectious diseases, which remain common in many areas of Latin America, further compromise neurological development and may aggravate seizure activity [107].

Compounding these issues are broader demographic and public health challenges. Despite efforts at family planning, birth rates remain high in some rural communities, putting additional pressure on already overstretched healthcare systems. Furthermore, increases in substance abuse—including alcohol and illicit drugs—among adolescents in some countries have introduced new risk factors for seizure development and reduced treatment adherence [108, 109].

Efforts to bridge these disparities remain fragmented. Some community‐based cooperatives and nongovernmental organizations offer assistance with medication costs and epilepsy education, but these efforts are not integrated into national public health strategies. The lack of national and regional epilepsy registries also hampers the development of efficient care protocols, making it challenging to allocate resources or evaluate intervention outcomes [102].

Ultimately, the management of CRE in Latin America cannot be separated from the socioeconomic realities that define healthcare access in the region. Addressing the disease requires not only medical innovation but also public policies that strengthen health systems, improve access to care, and reduce stigma. Targeted investment in diagnoses, medication access, specialist training, and patient education is essential to reducing the burden of RE and improving outcomes for affected children across Latin America.

### 3.1. Nondrug Treatment

Currently, various nondrug therapies have been used to treat RE, but the recommended treatment depends on each patient. Therefore, once drug resistance is detected, a specialized evaluation is recommended [103].

Therapy for RE involves surgical intervention, known as brain resective surgery, in which the region of the brain causing the epilepsy is removed. This procedure is among the most effective, and over the years, the technique has been refined to reduce risks and increase effectiveness; however, in Latin America, there are not enough operating rooms or specialists to perform this surgery. In general, this procedure is the most recommended when the area of the brain causing the epilepsy is safe to remove, with more than 50% of patients showing a complete recovery (in developing countries). In comparison, less than 10% of patients achieve a full recovery using only drugs when drug resistance has been observed. Even when brain resective surgery fails to eradicate seizures, the frequency is drastically reduced, thus improving the patient′s quality of life [110]. Unfortunately, this procedure is highly invasive and expensive, making it inaccessible to low‐income individuals and less widely available in developing countries such as Latin America. Another limitation is that it cannot be performed if the epilepsy‐causing area is in a problematic or compromised area. Furthermore, surgery can cause changes in the neuronal network, such as an injury, so it carries a higher risk than other procedures [111].

An alternative therapy that has emerged in recent years is laser interstitial thermal therapy (LITT). This therapy consists of a neurosurgical procedure that uses a laser to generate heat to directly target epileptogenic tissue, thereby destroying hyperexcitable neurons in the area causing the epilepsy [112]. This therapy is a minimally invasive procedure with a short recovery time, but its effectiveness is lower than surgical methods, with a success rate of 52.5% 2 years after treatment [113]. Other studies have shown that the procedure′s effectiveness decreases over time, and compared with surgical methods, seizure freedom declines faster after LITT. Specifically, the seizure‐free rate 1 year after LITT was found to mirror that of 5–10 years after brain surgery [103]. However, this technique has several limitations, including its cost. LITT is not common in developing countries [114].

## 4. Sol‐Gel–Derived Nanoparticles: Chemistry and Properties

Although a wide range of nanoparticle systems—including dendrimers, solid lipid nanoparticles (SLNs), nanostructured lipid carriers (NLCs), and polymeric nanoparticles—have demonstrated potential for enhancing AED delivery, they are not without critical limitations. Issues such as low drug‐loading capacity, instability during storage, batch‐to‐batch variability, and difficulties in controlling release kinetics hinder their clinical translation. Furthermore, ensuring uniform dispersion of active pharmaceutical ingredients and achieving sterile, biocompatible formulations suitable for pediatric neurological applications remains a formidable challenge. In this context, sol‐gel–derived nanoparticles offer an up‐and‐coming and versatile alternative. Developed via a well‐established chemical process, these inorganic and hybrid nanomaterials offer unique advantages in structural precision, compositional tunability, long‐term stability, and the integration of drug molecules into a three‐dimensional porous matrix. These properties make sol‐gel systems particularly attractive for controlled and sustained delivery of AEDs directly to the brain, especially in the management of CRE [115, 116].

### 4.1. Overview of Sol‐Gel Chemistry

The sol‐gel process is a bottom‐up synthetic route that transforms molecular precursors into solid nanostructures through a series of hydrolysis and condensation reactions (Figure 7). Typically, metal alkoxides such as tetraethyl orthosilicate (TEOS) for silica or titanium isopropoxide for titania, are used as starting materials. Under carefully controlled conditions involving water, alcohol‐based solvents, and acidic or basic catalysts, these precursors undergo hydrolysis, where alkoxide groups are replaced by hydroxyl groups, followed by polycondensation, which results in the formation of M–O–M (metal–oxygen–metal) linkages and the evolution of a three‐dimensional gel network [117, 118].

**Figure 7:**
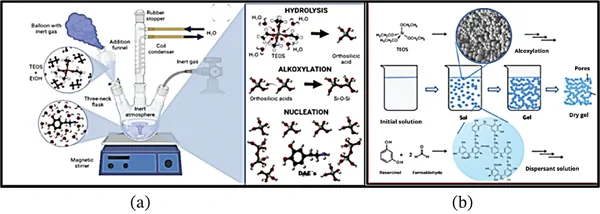
Sol‐gel technology. (a) Reactor (inert atmosphere). (b) Products after gelation. Figure made in BioRender.com (accessed on October 25, 2025).

This transformation from a liquid “sol” to a semisolid “gel” marks the critical step at which drug molecules can be integrated, or “co‐gelled,” into the developing matrix. The gel is then subjected to drying and aging steps to yield xerogels or aerogels with tuneable porosity and structural properties. These matrices can be processed into nanoparticles, monoliths, fibers, coatings, or injectable gels, depending on the intended clinical application [119].

### 4.2. Physicochemical Properties Relevant to Drug Delivery

One of the most significant advantages of sol‐gel–derived nanoparticles is their ultrafine, monodisperse nature. These particles typically range between 1 and 100 nm in size, allowing them to penetrate the BBB and distribute efficiently within brain tissues [15]. Furthermore, the porous internal structure of sol‐gel materials enables high drug loading and sustained release, a crucial feature for maintaining consistent therapeutic levels of AEDs while minimizing systemic exposure and side effects [120, 121].

Unlike other nanoparticle systems that encapsulate drugs in external shells or lipid bilayers, sol‐gel systems enable uniform molecular dispersion of drugs within the nanoporous network. It leads to superior control over release kinetics and reduces the risk of premature degradation.

Quantum mechanics and density functional theory (DFT) are based on an approximation that considers all properties of the ground states to be functional of the density:

aA+bB+⋯⟶sS,

rA=−dNAdT,rB=−dNBdT,rC=dNCdT.

A nanoparticle synthesized with sol‐gel has first‐order kinetics. It has the following kinetic behavior, reaction rate, and

−1adNadT=1qdNQdT.

Generalizing for N chemical species participating in M independent reactions

∝i1A1+∝i2A2+⋯+∝iNAN=0,

or in closed form

∑i=1N∝iδAδ=0123 i=,,,⋯,M.

When there is only one reaction, the *δ*_i1_ index i is inconsistent. It is positive for the reaction products and negative for the reactants. Reaction rates are generally expressed as intensive variables, for example, mass or volume. That is,

r=−1aVdNadt=−1aVddtCAV,=−1aVVdCAdt+CAdVdt

If the volume is constant,

dVdt=0,

and

r=−1aVVdCAdt=−1adCAdt.

Conversion and reaction progress

XA=NA0−NANA0,Xδ=Nδ0−NδNδ0,

l=Nj−Nj0∝j,

or also

Nj=Njl+Nj0,

where *l* is the reaction rate and is the same for all reaction species. For multiple reactions,

Nj=Nj0+∑i=1M∝ijli.

The order of a reaction considering the reaction

A+B⟶P,

r=kcCAaCBb,

kc=reaction coefficient,

a=order in reactant a,

b=order in reactant b,

a+b=total order of the reaction=1.

Significantly, the release rate can be modulated by tuning the physicochemical parameters of the sol‐gel process, including the water‐to‐alkoxide ratio, solvent polarity, gelation pH, and catalyst type. These variables influence pore size, surface area, network density, and the degree of hydroxylation, which in turn determine the diffusion‐based or degradation‐based release mechanisms of the encapsulated drug (Table 4) [122, 123].

**Table 4:** Reported textural properties and porosity of functionalized nanostructures.

Sample	*S*_*B* *E* *T*_(*m* ^2^/*g*)	*V* *p*(*c* *m* ^3^/*g*)	*D* *p*(Å)
TiO_2_ − pH = 12 (2020/578)	494	0.33	27
TiO_2_ − DPH − 50	497	0.55	41
TiO_2_ − DPH − 100	—	—	—
TiO_2_ − DPH − 150 (2020/574)	530	0.67	51
TiO_2_ − DPH − 200	526	0.59	45
TiO_2_ − DPH − 250	530	0.59	120
TiO_2_ − DPH − 1000	300	—	—
TiO_2_ − VPA − 200 (2020/584)	439	0.38	35
TiO_2_ − VPA − 400 (2020/586)	609	0.61	40
TiO_2_ − VPA − 600 (2020/588)	598	0.32	110
TiO_2_ − VPA − 800 (2020/7/241)	509	0.48	38
TiO_2_ − VPA − 1000 (2020/583)	501	0.44	36

### 4.3. Biocompatibility and Sterilization

For pediatric neurological applications, biocompatibility and sterility are nonnegotiable prerequisites. Sol‐gel–derived materials, particularly silica and titania, have been shown to exhibit excellent biocompatibility with neural tissues [124–126]. Silica, for example, is bioinert and has been used safely in multiple biomedical devices. Furthermore, the low‐temperature nature of sol‐gel synthesis is compatible with therapeutically sensitive compounds, and the final product can be easily sterilized without compromising its structural integrity or drug payload [15, 127, 128]. It contrasts with lipid‐based nanoparticles or polymeric systems, which may degrade at elevated temperatures or undergo morphological changes during sterilization, limiting their scalability and shelf‐life [129, 130]. Additionally, sol‐gel–derived nanoparticles offer long‐term stability at ambient conditions, reducing logistical challenges of storage and transportation—especially critical in low‐resource healthcare settings common across Latin America [131].

### 4.4. Functionalization and Targeted Delivery

Another remarkable feature of sol‐gel nanoparticles is their amenability to surface functionalization. The silanol (Si–OH) or titanol (Ti–OH) groups present on the particle surface can be chemically modified with a variety of ligands [132, 133], including peptides [134–136], antibodies, or small molecules that target receptors overexpressed in epileptic foci [137, 138]. This capability opens the door to site‐specific delivery, whereby AEDs are preferentially released in regions of the brain most affected by seizure activity, such as the temporal lobe or hippocampus (Figure 8).

Figure 8FESEM images of (A) NP, (B) higher amplification, cryo‐FESEM images of (C) NP dissolved and vitrified in H_2_O, and (D) higher magnification. (E) SEM images of nanoporous TiO_2_ aggregates, which contain encapsulated valproic acid, dried at room temperature under vacuum (10 kPa). (F) Nitrogen adsorption–desorption isotherms at 77 K for the titania nanostructured particles at different concentrations of VPA as follows: reference–0, conc. 1–6.05, conc. 2–12.11, and conc. 3–30.3 mg VPA/g TiO_2_. (G) VPA adsorption (solid line)–desorption (short dotted line) kinetics and (H) parabolic diffusion reverse release model of 6.05 (blue solid line, curve 1) and 12.11 (green solid line; curve 2) mg of PA/g TiO_2_, in 30 mL of solvent (ethanol). (I) Dissolution–diffusion kinetic models of (a) zero‐order, (b) first‐order, and (c) modified Freundlich model for VPA release from VPA‐titania matrix. The linear modeling is shown as the solid curve, and the experimental, dynamic release, which is similar to the released drug, should be consumed by the metabolic process. The points are as follows: (1) 30.3, (2) 12.11, and (3) 6.05 mg VPA/g TiO_2_ to methanol over 800 h.

**Figure:**
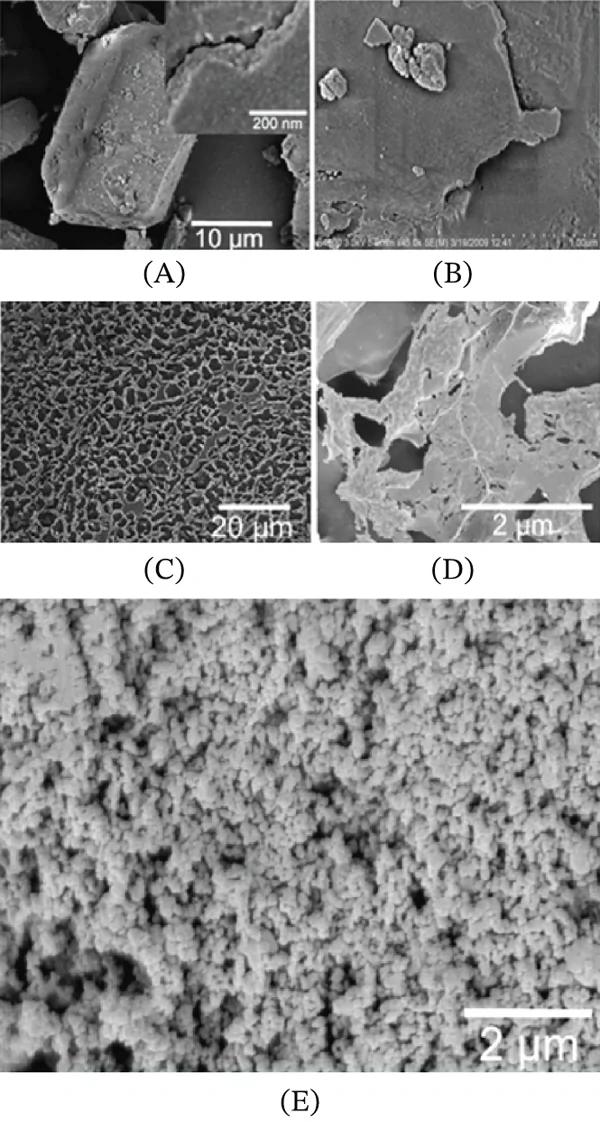

**Figure:**
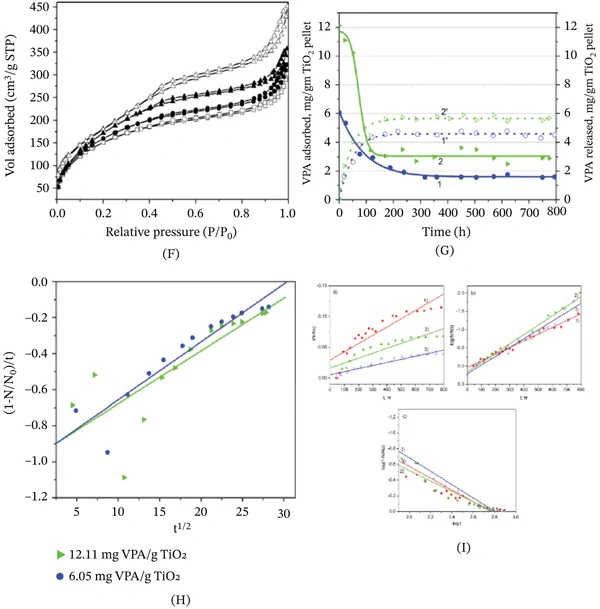

In addition, external stimuli‐responsive formulations can be engineered that trigger release in response to changes in pH, temperature, or enzymatic activity [139–142]—adding a layer of precision and adaptability to therapy. Such features are up‐and‐coming in RE, where localized and sustained drug release may significantly improve seizure control while minimizing adverse neurodevelopmental effects [143].

### 4.5. Current Limitations and Translational Challenges of Sol‐Gel Nanoparticle Systems

Despite their considerable potential for controlled AED delivery, sol‐gel–derived nanoparticles remain at a relatively early stage of clinical translation. Their successful implementation in pediatric epilepsy will depend not only on demonstrating therapeutic efficacy, but also on addressing several biological, technological, and regulatory challenges that remain insufficiently understood.

Although silica‐ and titania‐based materials are generally considered biocompatible, their biological behavior is strongly influenced by physicochemical characteristics such as particle size, morphology, surface charge, porosity, degradation rate, and surface functionalization. These parameters determine cellular uptake, biodistribution, and clearance, and may also influence the induction of oxidative stress, inflammatory responses, or cytotoxicity under certain exposure conditions. Consequently, nanoparticle formulations intended for neurological applications require comprehensive toxicological characterization beyond conventional in vitro cytocompatibility assays.

For CNS applications, long‐term safety is particularly relevant. Unlike many conventional drug‐delivery systems, localized nanoreservoirs may remain within neural tissues for prolonged periods, making it essential to evaluate chronic tissue responses, including neuroinflammation, glial activation, foreign‐body reactions, and possible alterations in neuronal function. Likewise, the long‐term biodegradation products of inorganic matrices and their potential accumulation within the brain remain insufficiently characterized, particularly in pediatric populations where treatment may extend over many years.

Another important consideration is the interaction of nanoparticles with the immune system. Following administration, nanoparticles rapidly adsorb plasma proteins, forming a protein corona that modifies their biological identity and may influence biodistribution, cellular uptake, and clearance by the mononuclear phagocyte system. These interactions can reduce targeting efficiency and introduce significant inter‐patient variability, emphasizing the need for standardized surface engineering strategies capable of minimizing undesirable immune recognition.

Furthermore, although numerous preclinical studies have demonstrated encouraging results in animal models, translation to human disease remains challenging. Differences in brain anatomy, BBB physiology, immune responses, and disease heterogeneity limit direct extrapolation of preclinical findings. In addition, CRE encompasses multiple etiologies and epilepsy syndromes, suggesting that a single nanoparticle platform is unlikely to be universally applicable across all patient populations.

Taken together, these considerations indicate that future research should extend beyond demonstrating improved seizure control and focus on establishing long‐term safety, reproducible manufacturing, standardized characterization protocols, and robust translational evidence capable of supporting clinical implementation.

## 5. Localized Sol‐Gel Nanoreservoirs for AED Delivery: Evidence From Preclinical Models

Localized sol‐gel nanoreservoirs represent a distinct strategy within nanoparticle‐based AED delivery. Unlike systemic nanocarriers designed to circulate through the bloodstream and cross the BBB, localized nanoreservoirs are conceived as implantable or site‐specific matrices capable of releasing AEDs directly within or near the epileptogenic focus. This approach is particularly relevant for focal forms of RE, where seizure activity originates from anatomically defined brain regions and where sustained local drug exposure may provide therapeutic advantages over repeated systemic administration.

The rationale for this strategy is based on three interconnected principles. First, conventional AED therapy often requires long‐term or lifelong administration, making therapeutic adherence, dose adjustment, and cumulative toxicity major concerns. Second, systemic administration exposes the entire organism to the drug, even though only a fraction of the active compound reaches the epileptogenic tissue. Third, localized controlled‐release systems may maintain more stable drug concentrations at the target site while reducing systemic exposure and potentially decreasing dosing frequency.

Within this context, sol‐gel–derived silica and titania matrices offer several advantages for AED delivery, including structural stability, tunable porosity, high drug dispersion, and the possibility of modulating release kinetics through changes in synthesis parameters. The following sections summarize the molecular rationale, material design, and preclinical evidence supporting the use of sol‐gel nanoreservoirs for localized AED delivery, with particular emphasis on temporal lobe epilepsy (TLE) as a proof‐of‐concept model.

### 5.1. Molecular Design of Sol‐Gel Nanoreservoirs: Insights From DFT

Controlled drug delivery depends fundamentally on the physicochemical interactions established between the therapeutic molecule and the carrier matrix. Drug adsorption, encapsulation efficiency, release kinetics, and long‐term stability are ultimately governed by molecular‐level interactions that cannot be directly observed experimentally. Consequently, theoretical approaches based on DFT provide an important framework for understanding and predicting drug–matrix interactions before nanoparticle synthesis.

Using both classical and quantum‐mechanical methods, models have been developed to understand the atomic and electronic structure of materials used as matrices under various conditions arising from synthesis, such as OH saturation (Figure 9). Furthermore, calculations have been performed on some characteristic molecules of the drugs used in these treatments to determine their physicochemical properties and establish the potential for no disturbance in an environment such as that obtained in matrices synthesized by the sol‐gel method.

**Figure 9:**
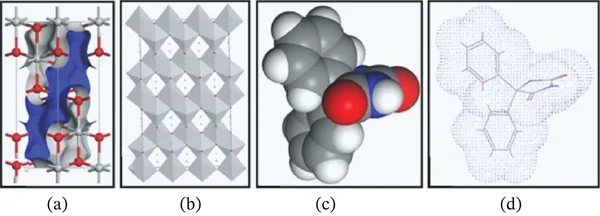
Structure generated with a 20% OH saturation in a titania material in the anatase phase, both in its (a) unit cell model with the exemplified charge distribution, and as a (b) superlattice that defines the intracrystalline pore sizes and (c) the atomic distribution and (d) electron density scheme in the characteristic phenytoin molecule.

DFT encompasses a family of methodologies for quantum‐mechanical electronic‐structure calculations with broad applications to organic and main‐group molecules, as well as to more complex systems (Figure 10).

**Figure 10:**
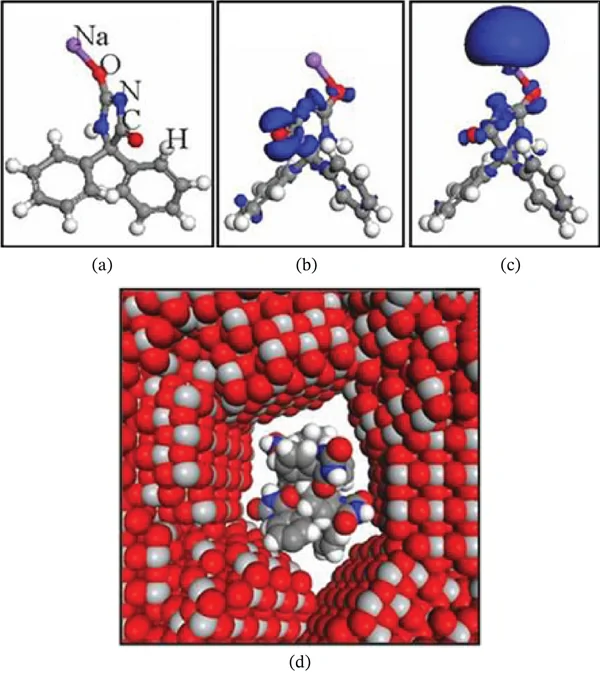
(a–c) Nanoparticles gelled with sodic phenytoin, DFT simulation of the reservoir, and electronic distribution. (d) DFT simulation of the controlled release of sodic phenytoin.

These methods are particularly valuable for transition‐metal complexes, where electron‐correlation effects can be substantial. Problems that can be effectively studied include electronic structure, charge and spin distributions, molecular geometries, and the energies of HOMO and LUMO orbitals. DFT was used to calculate the energy distribution in a titania nanoreservoir containing phenytoin sodium, as well as in titania containing valproic acid. DFT is among the most common methods for theoretical simulation of electronic structure. Its foundations rest on the determination of the ground state of electrons in a system from the time‐independent many‐body Schrödinger equation:

Pio=Pioio2⋯onorte,

HPi∧=T∧+V∧+UPi∧=∑i=1norte−h2metroi∇i2+∑i=1norteVoi+∑i<1norteUoioyoPi=miPi,

Etρ=Tρ+Uρ+Excρ,

Coulomb energy *E*_*x* *c*_[*ρ*].

Chemical potential is defined as the sum of the electronegativity of the atoms in the system, which gives us the total negative charge. The total positive charge is the sum of the electropositivity of each atom.

*T*[*ρ*]_[*Δ*]_ is the kinetic energy of the particles with electrostatic force:

μ=∂E∂Nv=−χ,

where *E* is the energy, and *N* is the number of electrons in the system at a constant volume *v*. The hardness of the material can be expressed with the following equation:

η=12∂μ∂Nv=12∂2μ∂N2v.

This implies, with respect to the front orbitals, an expression of the type:

u=εHOMO+εLUMO2,

η=εHOMO−εLUMO2.

At the boundary of molecular orbitals (HOMO) and (LUMO), it can be observed that the behavior of the charge distribution is totally different in the framework that has hydroxyl groups compared with that without hydroxyls. It can be interpreted as a function of the greater affinity and chemical selectivity that the structure may exhibit for different neurological drugs, depending on the extent of their hydroxylation.

### 5.2. Design and Optimization of Sol‐Gel Nanoreservoirs

The successful development of localized controlled‐release systems requires careful optimization of both the inorganic carrier and the drug payload. Unlike conventional pharmaceutical formulations, in which the active ingredient is simply dispersed within an excipient, sol‐gel nanoreservoirs are designed to integrate the therapeutic molecule into the three‐dimensional oxide network during synthesis. Consequently, the physicochemical properties of the final material—including porosity, surface area, matrix density, and surface chemistry—directly determine encapsulation efficiency, release kinetics, and biological performance.

To identify formulations suitable for intracerebral implantation, previous studies systematically evaluated silica‐ and titania‐based matrices containing different concentrations of phenytoin sodium and valproic acid [144, 145]. Both monolithic and nanostructured sol‐gel systems were investigated to determine the influence of matrix composition on biocompatibility and drug‐release behavior. These studies demonstrated that sol‐gel synthesis permits homogeneous molecular dispersion of AEDs throughout the oxide network while preserving the structural integrity of both the matrix and the encapsulated drug.

Formulation optimization involved evaluating multiple combinations of oxide composition and drug concentration in order to identify systems capable of providing sustained release without inducing local tissue toxicity or proconvulsant effects. As summarized in Table 5, both silica‐ and titania‐based reservoirs exhibited excellent biocompatibility over a broad range of formulations. However, only specific drug concentrations produced favorable biological responses, illustrating the importance of carefully balancing drug loading with release kinetics.

**Table 5:** List of synthesized nanostructured reservoirs.

Reservoir	Conc. of Ph (mg)	VPA conc. (mg)	Biocomp/Conduct
TiO_2_‐sol‐gel (titania)	0	0	Biocompatible/normal
SiO_2_‐sol‐gel (silica)	0	0	Biocompatible/normal
TiO_2_‐nanotubes	0	0	Biocompatible/normal
SiO_2_‐ordinate	0	0	Biocompatible/normal
SiO_2_‐sol‐gel monoliths	0	0	Biocompatible/normal
**Evidence with epileptic rats**			
SiO_2_‐monolith‐sol‐gelPh‐0.005	0.005	0	Biocompatible/convulsant
SiO_2_‐monolith‐sol‐gel‐Ph‐0.01	0.01	0	Biocompatible/not tested
SiO_2_‐monolith‐sol‐gel‐Ph‐0.1	0.1	0	Biocompatible/convulsant
SiO_2_‐monolith‐sol‐gel‐Ph‐1	1	0	Biocompatible/not tested
SiO_2_‐monolith‐sol‐gel‐Ph‐5	5	0	Biocompatible/does not cause seizures
SiO_2_‐monolith‐sol‐gel‐Ph‐10	10	0	Biocompatible/does not cause seizures
SiO_2_‐sol‐gel‐Ph‐25	1.25	0	Biocompatible/convulses
SiO_2_‐sol‐gel‐Ph‐50	2.5	0	Biocompatible/not tested
SiO_2_‐sol‐gel‐Ph‐100	5	0	Biocompatible/does not cause seizures
SiO_2_‐sol‐gel‐Ph‐150	7.5	0	Biocompatible/not tested
SiO_2_‐sol‐gel‐Ph‐200	10	0	Biocompatible/does not cause seizures
SiO_2_‐sol‐gel‐Ph‐250	12.5	0	Biocompatible/convulses
SiO_2_–sol‐gel‐1000	50	0	Biocompatible/convulses
TiO_2_‐sol‐gel‐Ph‐1	0.05	0	Biocompatible/convulses
TiO_2_‐sol‐gel‐Ph‐10	0.5	0	Biocompatible/not tested
TiO_2_‐sol‐gel‐Ph‐20	1	0	Biocompatible/not tested
TiO_2_‐sol‐gel‐Ph‐50	2.5	0	Biocompatible/convulses
TiO_2_‐sol‐gel‐Ph‐100	5	0	Biocompatible/does not cause seizures
TiO_2_‐sol‐gel‐Ph‐150	7.5	0	Biocompatible/does not cause seizures
TiO_2_‐sol‐gel‐Ph‐200	10	0	Biocompatible/not tested
TiO_2_‐sol‐gel‐Ph‐250	12.5	0	Biocompatible/not tested
TiO_2_‐sol‐gel‐Ph‐1000	50	0	Biocompatible/not tested
TiO_2_‐sol‐gel‐VPA‐10	0	0. 5	Biocompatible/convulses
TiO_2_‐sol‐gel‐VPA‐20	0	1	Biocompatible/not tested
TiO_2_‐sol‐gel‐VPA‐50	0	2.5	Biocompatible/not tested
TiO_2_‐sol‐gel‐VPA‐100	0	5	Biocompatible/convulses
TiO_2_‐sol‐gel‐VPA‐200	0	10	Biocompatible/does not cause seizures
TiO_2_‐sol‐gel‐VPA‐400	0	20	Biocompatible/does not cause seizures
TiO_2_‐sol‐gel‐VPA‐600	0	30	Biocompatible/does not cause seizures
TiO_2_‐sol‐gel‐VPA‐800	0	40	Biocompatible/not tested
TiO_2_‐sol‐gel‐VPA‐1000	0	50	Biocompatible/convulses
SiO_2_‐ord‐Ph‐5	0.5	0	Biocompatible/not tested
SiO_2_‐ord‐Ph‐200	20	0	Biocompatible/does not cause seizures
SiO_2_‐ord‐Ph‐1000	100	0	Biocompatible/not tested
SiO_2_‐ord‐VPA‐100	0	10	Biocompatible/not tested
SiO_2_‐ord‐VPA‐200	0	20	Biocompatible/does not cause seizures

The comparative evaluation of these formulations also demonstrated that release behavior depends not only on the quantity of drug incorporated into the matrix but also on the physicochemical characteristics of the carrier itself. Parameters such as pore architecture, hydroxylation degree, and oxide composition influence drug adsorption and diffusion through the porous network, ultimately determining the duration and stability of drug release. Consequently, rational optimization of the carrier material is as important as selection of the therapeutic agent itself.

Although these studies primarily served as proof‐of‐concept investigations, they established the feasibility of engineering sol‐gel nanoreservoirs with tunable physicochemical properties capable of supporting localized, sustained delivery of conventional AEDs. These findings provided the experimental foundation for evaluating localized implantation strategies in preclinical models of focal epilepsy.

### 5.3. Why TLE?

Among the various forms of CRE, TLE represents one of the best characterized focal epilepsy syndromes and remains the most extensively investigated experimental model for evaluating localized therapeutic interventions. Unlike generalized epilepsies, where epileptic activity involves widespread neuronal networks, seizures in TLE originate predominantly from anatomically defined structures, including the hippocampus, amygdala, and adjacent temporal lobe regions. This focal organization makes TLE particularly suitable for assessing site‐specific drug‐delivery strategies.

Mesial TLE is characterized by progressive neuronal loss, reactive astrogliosis, hippocampal sclerosis, and network reorganization, all of which contribute to recurrent seizure generation and pharmacoresistance [146]. Magnetic resonance imaging frequently demonstrates hippocampal atrophy and signal alterations that facilitate localization of the epileptogenic focus, allowing accurate correlation between structural abnormalities and seizure origin.

From the perspective of drug delivery, the existence of a well‐defined epileptogenic focus provides an important experimental advantage. Localized therapies can be directed precisely toward the region responsible for seizure initiation, allowing investigators to evaluate whether sustained local drug release offers therapeutic benefits beyond those achieved through systemic administration. Consequently, TLE has become the preferred proof‐of‐concept model for investigating implantable controlled‐release systems before considering their application to other forms of RE.

Importantly, the objective of these studies is not to suggest that localized nanoreservoirs should replace systemic pharmacotherapy in all patients with epilepsy. Rather, TLE provides an experimentally reproducible platform for evaluating whether localized sustained drug delivery can overcome some of the principal limitations associated with conventional AED administration, including poor brain bioavailability, fluctuating drug concentrations, systemic toxicity, and limited therapeutic adherence

## 6. Intranasal Administration of NP With AEDs

Intranasal administration is a noninvasive and immediate route for drug delivery to the brain [147]. Transport from the nose to the CNS occurs through two neuronal routes by axonal transport: the olfactory and trigeminal pathways. The first occurs through the olfactory sensory neurons of the olfactory nerve, located in the olfactory epithelium of the upper part of the nasal cavity, passing through the cribriform plate and reaching the olfactory bulb [148]. The second is through the trigeminal nerve, which has ophthalmic, maxillary, and mandibular branches that connect to the trigeminal ganglion, which enters the CNS and connects to the brainstem and spinal cord [149]. In this way, deeper brain regions are reached, facilitating the delivery of controlled‐release AEDs at a much higher rate than traditional methods [150].

Drug delivery occurs in three ways: intracellularly, where nanoparticles containing AEDs are internalized by endocytosis in olfactory neurons, then transported to the trigeminal nerve and subsequently released by exocytosis at their projection site. The second is through the paracellular route in the cranial nerves: The drug travels from the nasal cavity to the lamina propria via the olfactory epithelium and tight junctions to the perineural space and, by perivascular pumping, it reaches the subarachnoid space, finally reaching brain tissue. The third is when the drug passes through the cells via transporters or endocytosis via the adsorptive transcytosis mechanism and is called transcellular. In the latter, there is an interaction between the positively charged NP ligand and the negative charge of the cell membrane (Figure 11) [151–154].

**Figure 11:**
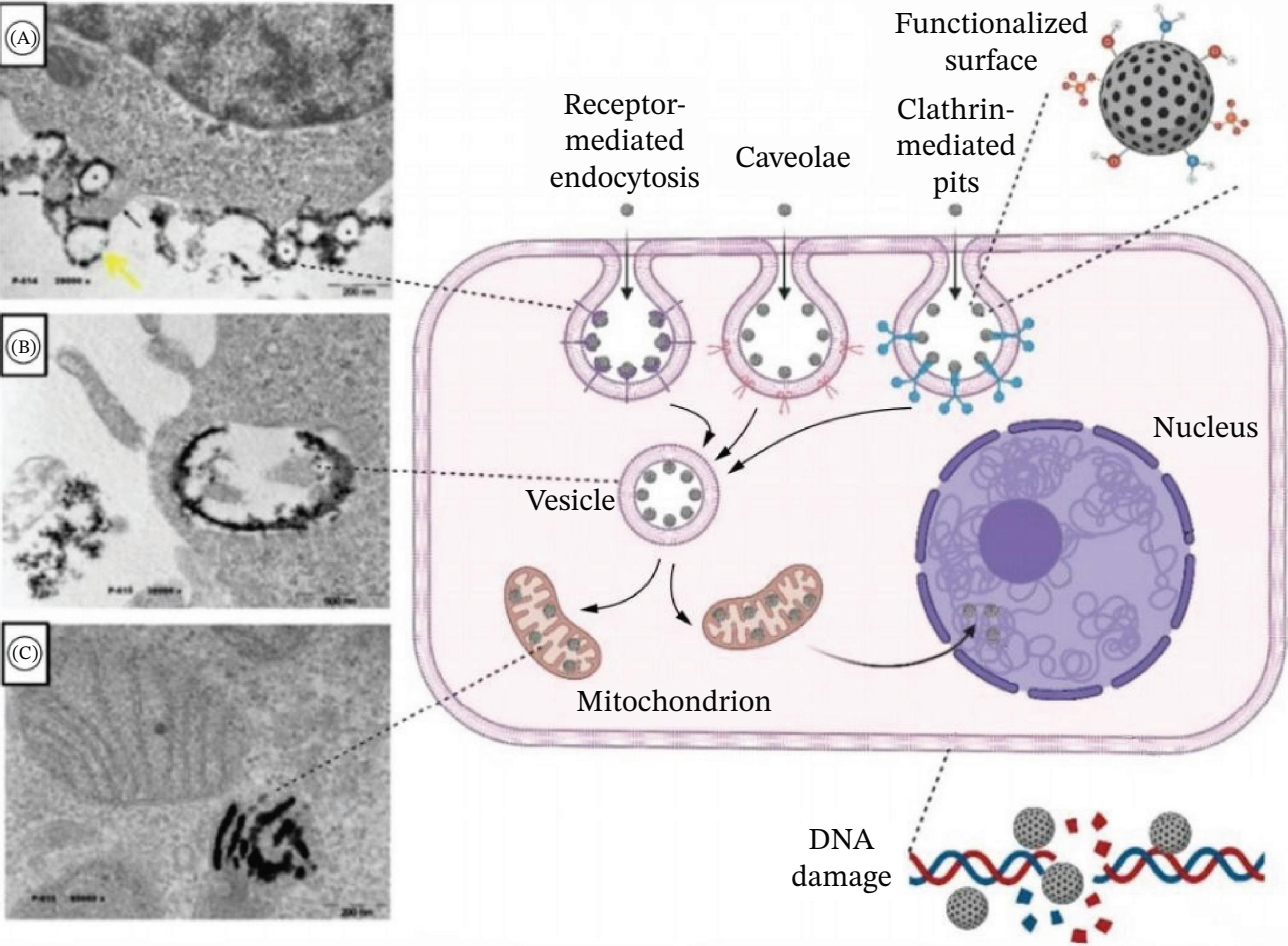
Ligand–receptor mediated endocytosis: selective internalization mechanism of nanoreservoirs + antiepileptic. (A) When in the presence of nanoreservoirs, specific receptors interact with the superficial coating on the surface of the NPs (the ligands) and carry out a pinocytosis‐like process (yellow and black arrows). (B) Invagination of the membrane allows for nanoreservoir internalization. (C) When the vacuole breaks, part of the nanoparticles fills the mitochondria (transmission electron micrograph taken at our laboratory). Figure 11 made in BioRender.com (accessed on October 25, 2025).

The use of nanoparticles for more efficient delivery of AEDs via the nose–brain route has been reported in the literature. In mice, oxcarbazepine‐loaded poly (lactic‐co‐glycolic acid) (PLGA) nanoparticles have been used to control epileptic seizures, reducing the administration dose to once every 24 h [155]. The research team of Hou et al. [156] synthesized a coreshell system (D‐T7/Tet1‐lipids@PL) of nanoparticles using a microfluidic chip for the delivery of lamotrigine (LTG), demonstrating precise targeting of the drug to neurons. It resulted in a highly positive therapeutic effect on ER, observed in murine species. El‐Nahas et al. [157] fabricated berberine‐loaded hyaluronic acid (HA)–based zein (a protein from corn) nanoparticles to treat neurodegeneration in pilocarpine: C_11_H_16_N_2_O_2_ models of epilepsy. HA is reported to be crucial in the binding between the drug and CD44 receptors in the hippocampus of individuals with frontal lobe epilepsy. Wong et al. [158] proposed an oxytocin‐encapsulating nanoparticle to control induced seizures and improve social behavior in RH/+ mutant mouse models, which are exemplary of Scn1alpha‐derived epilepsy, the alpha subunit of the sodium channel type 1 protein. Jain et al. [159] formulated a zonisamide‐loaded nanoemulsion that improves cerebral uptake, solubility, stability, and compatibility with the nasal mucosa. Wang et al. [160] developed a PEG‐PLA nanoparticle modified with the angiopep‐2 peptide for the targeted delivery of ketoconazole to improve treatment efficiency for RE. Decreased expression of pregnane X receptor (PXR) and its downstream proteins was observed, reducing seizures in patients. Shu et al. [161] created a PEG‐PLA nanoparticle (TPnHESR@CBZ) functionalized with peptides, which allows it to pass through the nasal epithelium, maintain a specific neuronal orientation, and release CBZ upon detecting abnormal brain electrical activity. It reduced the severity and duration of seizures. The group of Zhou et al. [162] developed a polymeric nanomicelle (LTG@DPLB) functionalized with dehydroascorbic acid, which directs the system to the GLUT1, and loaded with LTG. Its core, made of polymeric chains of phenylpropanol ester, was reported to be sensitive to ROS, so when high concentrations were detected, the micelle eliminated them and stabilized glial cells in the brain microenvironment, mitigating epileptic seizures and their sequelae. Nogueira et al. [163] describe the use of a cationic nanoemulsion (BCP‐NE) loaded with beta‐caryophyllene (BCP) with high residence time in the nasal mucosa, 98% encapsulation efficiency, and anticonvulsant activity in delaying tonic‐clonic seizures. In this sense, nanoparticle functionalization is vital, as it enhances drug performance after application.

### 6.1. Limitations of Conventional Drug Delivery in Epilepsy

The pharmacological management of epilepsy involves long‐term administration of AEDs to suppress abnormal electrical discharges in the brain. However, many of these drugs have poor pharmacokinetic profiles. For instance, compounds like valproic acid, CBZ, and phenytoin have low aqueous solubility and undergo significant first‐pass metabolism, which reduces their bioavailability and necessitates higher or more frequent dosing [164]. Additionally, many AEDs are substrates for efflux transporters, such as P‐gp, which actively pump drugs out of the CNS, further limiting their therapeutic concentrations in the brain [165].

It is especially problematic in children, whose metabolic rates and body composition differ from those of adults, affecting drug absorption and distribution. Furthermore, prolonged exposure to high systemic doses can lead to significant adverse effects, including hepatotoxicity, sedation, cognitive impairment, behavioral disturbances, and endocrine dysfunction [166]. These limitations underscore the urgent need for improved drug delivery methods that can enhance CNS targeting while minimizing systemic toxicity.

### 6.2. Nanoparticles as Drug Delivery Vehicles

Nanoparticles are colloidal carriers ranging from 1 to 100 nm in size and composed of materials such as lipids, polymers, metals, or proteins [167, 168]. Their small size and surface‐modifiable properties enable high control over drug release profiles, biodistribution, and cellular uptake. By encapsulating or conjugating AEDs within nanoparticles, it becomes possible to enhance drug solubility, protect the active compound from premature degradation, and achieve targeted delivery to specific brain regions affected by epileptic activity [169].

One of the most compelling advantages of nanoparticles is their ability to cross the BBB. This highly selective barrier typically restricts the entry of large or hydrophilic molecules into the brain [170]. Nanocarriers can be engineered to exploit receptor‐mediated transcytosis pathways or to evade efflux mechanisms, thereby improving CNS drug delivery. Furthermore, by modifying the surface of nanoparticles with ligands such as antibodies, peptides, or polysaccharides, it is possible to direct them toward epileptogenic foci or cell types involved in seizure generation and propagation [171, 172].

### 6.3. Dendrimers

Among the most promising nanocarrier systems for treating epilepsy are dendrimers. These are highly branched, tree‐like synthetic macromolecules with a well‐defined, monodisperse structure and multiple surface functional groups [173]. Polyamidoamine (PAMAM) dendrimers, in particular, have been widely studied for CNS applications due to their biocompatibility, internal cavities suitable for drug encapsulation, and modifiable surfaces for targeted delivery [174]. Dendrimers have demonstrated effectiveness in preclinical epilepsy models [175]. For example, CBZ, a widely used AED, has been successfully loaded into PAMAM dendrimers, resulting in improved drug solubility, controlled release, and reduced neurotoxicity [176]. These systems maintain sustained CNS drug levels, which can reduce dosing frequency and minimize fluctuations in plasma drug concentration—an important consideration for preventing breakthrough seizures. Additionally, dendrimers can be engineered to release their drug cargo in response to specific stimuli (e.g., pH and enzymes), offering precision in timing and location of drug action [177].

### 6.4. Lipid‐Based Nanoparticles

Lipid‐based nanoparticles, including SLNs and NLCs, have also gained attention as effective drug delivery systems for epilepsy [178]. These carriers are composed of biocompatible lipids that can encapsulate lipophilic drugs like valproic acid, diazepam, and topiramate [179]. SLNs are composed of solid lipids that provide a rigid matrix for drug incorporation. In contrast, NLCs combine solid and liquid lipids to enhance drug loading capacity and reduce crystallinity, thereby improving release kinetics and stability. These lipid nanoparticles offer several advantages for epilepsy therapy. Their lipophilic nature facilitates interaction with cell membranes and enhances drug penetration across the BBB [180]. Once in the brain, the nanoparticles can provide sustained release of the encapsulated AED, maintaining therapeutic levels for extended periods. Furthermore, lipid carriers protect sensitive drugs from degradation and reduce systemic exposure, thereby lowering the risk of adverse effects [181].

Preclinical studies have shown that SLNs encapsulating valproic acid exhibit enhanced brain targeting and improved anticonvulsant activity compared with conventional formulations [182]. Similarly, NLCs loaded with diazepam have demonstrated superior bioavailability and extended sedation duration in animal models, suggesting potential for improved seizure control in patients with RE [149].

### 6.5. Polymeric Nanoparticles

Polymeric nanoparticles are another class of delivery vehicles that have been investigated for the treatment of epilepsy. These systems are typically composed of biodegradable and biocompatible polymers such as polylactic acid (PLA), polyglycolic acid (PGA), PLGA, or chitosan [183]. Polymeric nanoparticles can be designed for controlled, sustained drug release over hours or days, which is particularly useful for chronic conditions such as epilepsy.

One notable advantage of polymeric nanoparticles is their structural versatility. Drugs can be physically encapsulated within the polymer matrix or chemically conjugated to the polymer backbone. Furthermore, the surface of these nanoparticles can be functionalized with ligands that enable BBB crossing or targeting of specific brain regions [184]. Research involving PLGA nanoparticles loaded with LTG or topiramate has shown promising results in animal models, with improved pharmacokinetics, reduced dosing frequency, and enhanced seizure suppression compared with standard drug formulations [185]. Chitosan‐based nanoparticles, due to their mucoadhesive and permeation‐enhancing properties, have also been studied for intranasal delivery of AEDs, offering a noninvasive route that bypasses the BBB [186].

### 6.6. Other Nanocarrier Systems

In addition to dendrimers, lipid‐based systems, and polymeric nanoparticles, several other nanocarrier types have shown potential in preclinical epilepsy models. These include (i) liposomes: phospholipid vesicles that can encapsulate both hydrophilic and lipophilic drugs. Although less stable than other nanoparticles, they offer excellent biocompatibility and can be modified for targeted CNS delivery [187, 188]; (ii) nanoemulsions: oil‐in‐water or water‐in‐oil emulsions with droplet sizes in the nanometer range. They are beneficial for enhancing the solubility and bioavailability of poorly water‐soluble drugs [189, 190]; (iii) micelles: self‐assembling amphiphilic molecules that form core‐shell structures [191, 192]. Their small size and hydrophobic core make them ideal for delivering lipophilic AEDs. These alternative platforms continue to be refined and adapted for specific clinical scenarios, including epilepsy and other neurodegenerative or neuroinflammatory diseases.

## 7. Future Directions

Although most nanoparticle‐based therapies for epilepsy remain in preclinical stages, the accumulating body of evidence supports their potential as safer, more effective alternatives to traditional oral AEDs—particularly for patients with RE. Pediatric patients, who are often the most vulnerable and least responsive to conventional treatments, may especially benefit from nanoparticle‐mediated drug delivery due to reduced dosing requirements, better brain targeting, and minimized side effects. However, several challenges must still be addressed before these technologies can reach clinical application. These include concerns about long‐term safety, production scalability, storage stability, and regulatory approval. Ongoing research is focused on optimizing particle design, identifying reliable targeting ligands, and conducting rigorous toxicity studies. In the future, integrating nanotechnology with personalized medicine and neuroimaging may enable individualized treatment regimens that adapt to each patient′s unique disease profile and genetic background.

Currently, nonpharmacological therapies such as brain‐respecting surgery, neurostimulation, or LITT are inaccessible to most people with epilepsy, so the need for emerging therapies is high. One emerging therapy is the use of nanocarriers. Nanomedicine has experienced exponential growth over the last 20 years; however, for epilepsy, work continues to focus on murine models. Several studies have used various types of nanocarriers to treat epilepsy in mouse models, but the FDA has approved none. However, several laboratories are already in clinical protocol [193]. Systems using nanoliposomes to cross the BBB are opting for nasal‐brain delivery via the trigeminal nerve, ensuring that more than 60% of the drug reaches the epileptogenic site [194, 195]. Metallic nanoparticles have been used to regulate the abnormal electrical conduction that causes seizures by placing metals such as gold, silver, platinum, iron, zinc, and copper [196, 197].

Furthermore, polymeric nanoparticles are very effective in crossing the BBB and delivering the AED to the epileptogenic focus [198, 199]. SLNs are effective nanocarriers of AEDs such as pitavastatin for intranasal administration [200, 201]. The use of nanomaterials also enables controlled release via nanosystems that can be electrically stimulated using conductive polymers, or by temperature, pH changes, or the release of ROS [139].

### 7.1. Customization for Pediatric Use

Children with RE often require long‐term, low‐dose, and continuous delivery of medications to avoid seizure recurrence and cognitive decline. Sol‐gel systems can be tailored to these requirements [19]. Injectable formulations, for instance, allow for minimally invasive, localized implantation near seizure foci, where the nanoparticle matrix can act as a drug reservoir, gradually releasing AEDs over weeks or even months [202, 203]. This approach reduces the need for frequent dosing and enhances patient compliance—a crucial factor in pediatric epilepsy management.

Furthermore, sol‐gel matrices can be designed to codeliver multiple drugs with distinct release profiles, enabling polytherapy within a single formulation [204, 205]. It could be especially beneficial in complex cases like LGS or Dravet syndrome, where combination therapy is often necessary but challenging to maintain due to pharmacokinetic incompatibilities and side effects.

### 7.2. Addressing Global Health Gaps

Beyond their technical merits, sol‐gel–derived nanoparticles have significant implications for global health equity, especially in low‐ and middle‐income regions such as Latin America. These systems offer the potential to reduce healthcare costs by enabling sustained and effective seizure control, decreasing hospitalization rates, and minimizing the need for surgical interventions [206]. The stability and ease of formulation also support local production under resource‐constrained conditions, aligning well with decentralized healthcare delivery models. The ability to develop tailored therapies using abundant raw materials, such as silica precursors and ethanol‐based solvents, further enhances the feasibility of deploying sol‐gel technologies in under‐resourced clinical environments [207]. Thus, sol‐gel systems not only represent a scientifically advanced platform but also a pragmatic solution to the unmet needs of children who have RE in disadvantaged regions.

## 8. Applications in RE

The emergence of nanotechnology—particularly sol‐gel–derived nanoparticles—has introduced a transformative approach to the treatment of CRE, a condition notoriously resistant to conventional pharmacological strategies. As previously discussed, conventional AEDs are limited by their poor bioavailability, systemic toxicity, and low brain penetration. Sol‐gel technology enables the design of nanodelivery systems that address these limitations by enabling targeted, sustained, and biocompatible drug release within the CNS [208].

In recent years, several preclinical studies have demonstrated the potential of sol‐gel–derived nanocarriers to enhance the therapeutic efficacy of AEDs. These systems allow antiepileptic compounds to be uniformly dispersed at the molecular level within a porous, inorganic matrix, such as silica or titania [209]. Upon implantation or administration, the drug is released gradually through a combination of diffusion and degradation mechanisms, thereby avoiding the plasma concentration fluctuations typical of oral regimens [210]. This controlled‐release profile is particularly beneficial in pediatric patients, where dose stability is essential to avoid breakthrough seizures and minimize adverse neurodevelopmental effects.

One of the most promising applications involves the use of CBZ‐loaded silica nanoparticles fabricated via sol‐gel condensation [211]. In vivo studies using rodent models of TLE have shown that intracerebral or intranasal administration of these nanocomposites significantly reduced seizure frequency and duration compared with conventional formulations. Histopathological analysis also revealed reduced neuronal damage and gliosis in treated animals, supporting the notion that sol‐gel nanoparticles not only enhance drug delivery but may also modulate the local inflammatory and oxidative environment in the epileptogenic focus.

Similarly, valproic acid, a broad‐spectrum AED often limited by hepatotoxicity and teratogenic risk, has been encapsulated in titania‐based sol‐gel systems and evaluated in preclinical models [212]. These studies demonstrated improved bioavailability and CNS targeting, along with a marked reduction in peripheral organ toxicity. The ability of sol‐gel materials to reduce systemic exposure by enabling site‐specific drug deposition provides a compelling rationale for their integration into treatment protocols for highly drug‐resistant epilepsies.

Further work has focused on the codelivery of multiple drugs within a single sol‐gel matrix. Given that CRE often requires polytherapy to achieve partial seizure control, sol‐gel platforms have been engineered to release combinations of AEDs, such as LTG and levetiracetam, at differential rates [213]. It is achieved by tuning the matrix′s porosity, surface chemistry, and degradation kinetics during synthesis. Such customization could help overcome the pharmacokinetic incompatibilities of oral polytherapy while minimizing adverse interactions.

Importantly, the route of administration is another domain in which sol‐gel systems demonstrate versatility. Although most current studies rely on stereotactic implantation of nanoparticle gels near the seizure focus (e.g., hippocampus or temporal lobe), ongoing research is exploring intranasal and subdural delivery as less invasive alternatives [214]. Intranasal administration, in particular, exploits the olfactory and trigeminal neural pathways to bypass the BBB, offering a noninvasive route for delivering nanocarriers directly into the CNS [215]. In one study, sol‐gel–derived silica nanoparticles loaded with ethosuximide were administered intranasally to juvenile rats with generalized epilepsy, resulting in prolonged seizure suppression and enhanced drug localization within the cortex and thalamus.

Another promising avenue is the use of injectable sol‐gel formulations, which can form a drug‐loaded depot upon contact with physiological fluids [216]. These injectable matrices—formulated as sol precursors that gel in situ—have been successfully used to deliver topiramate and phenytoin in animal models, showing steady release over several weeks and sustained seizure suppression [122]. This approach is especially relevant for low‐resource settings, where poor treatment adherence due to access barriers or stigma is a significant challenge. Long‐acting depots could reduce the need for daily dosing, decrease healthcare dependence, and improve long‐term outcomes.

Beyond seizure control, sol‐gel nanoparticles are being explored for the codelivery of neuroprotective and anti‐inflammatory agents to modify the course of epileptogenesis. For example, curcumin or resveratrol—natural compounds with antioxidant and anti‐inflammatory properties—have been successfully cogelled with AEDs into hybrid silica matrices [217, 218]. These formulations are aimed not only at suppressing seizures but also at limiting the long‐term brain damage associated with chronic epilepsy, potentially improving cognitive outcomes in affected children.

Although most of these applications remain within the preclinical stage, initial safety profiles are encouraging. Sol‐gel matrices composed of silica and titania have shown high biocompatibility, low immunogenicity, and ease of sterilization—essential properties for pediatric neurological use [15, 219]. Furthermore, these systems demonstrate long‐term physicochemical stability at ambient conditions, facilitating storage and distribution in low‐ and middle‐income countries, where temperature‐controlled supply chains are often unreliable.

From a clinical translational perspective, early‐phase human trials will require standardization of nanoparticle synthesis, optimization of gelation and release kinetics, and robust evaluation of neurotoxicity and pharmacodynamics. Pediatric trials will also need to consider developmental differences in brain permeability, drug metabolism, and long‐term safety. Regulatory agencies are increasingly recognizing the potential of nanomedicine (10.3389/fmed.2025.1544393), and sol‐gel–based formulations may benefit from existing frameworks for drug‐device combination products.

In summary, sol‐gel nanoparticles represent a highly versatile and promising platform for the treatment of CRE. By enabling controlled, targeted, and multidrug delivery to the CNS, these systems may significantly improve seizure control while minimizing systemic toxicity and enhancing quality of life. Their modular design, long‐term stability, and adaptability to low‐resource settings further underscore their potential to address treatment gaps in Latin America and other underserved regions. Continued research, including clinical translation, is critical to unlocking the full therapeutic value of this innovative technology in pediatric neurology.

## 9. Regulatory and Translational Considerations

A crucial step in translating sol‐gel–derived nanoparticles into clinical therapies for CRE is navigating the complex interface among regulatory compliance, translational science, and scalable production. Although these nanoparticle systems have demonstrated substantial therapeutic potential in preclinical models, their clinical implementation demands strict adherence to safety protocols, manufacturing standards, and evidence‐based validation—particularly in pediatric populations where developmental vulnerability amplifies the need for precision and caution [220].

One of the primary regulatory challenges stems from the hybrid nature of sol‐gel nanoparticles, which combine the characteristics of both pharmaceuticals and medical devices. International regulatory bodies such as the FDA and the European Medicines Agency (EMA) typically classify these as combination products, with the primary mechanism of action determining whether they are regulated predominantly as drugs or devices [221]. In the case of sol‐gel formulations used to deliver AEDs, their primary function is pharmacological, and they are usually classified under drug regulations. However, when formulations are designed for implantation or modified for targeted release (e.g., pH‐ or enzyme‐responsive delivery), additional device‐related criteria apply [222].

Latin American regulatory agencies, such as COFEPRIS in Mexico and ANVISA in Brazil, are still in the early stages of developing formal frameworks for nanomedicines. At present, these agencies follow general guidelines for biologics and modified‐release drugs but lack specific procedures for evaluating nanoparticle‐enabled systems [223]. This regulatory lag may slow clinical translation unless harmonized efforts are undertaken to align Latin American frameworks with international standards, supported by regional capacity‐building in nanotoxicology, Good Manufacturing Practice (GMP) production, and bioequivalence testing [224].

Preclinical safety evaluation is another indispensable component of the translational pipeline. Regulatory approval for first‐in‐human trials requires comprehensive toxicological and pharmacokinetic characterization of nanoparticle formulations (Table 6). Key parameters include particle size distribution and aggregation tendencies, surface charge (zeta potential), drug release kinetics, and biocompatibility with neural tissue. It is essential to determine biodistribution patterns and clearance rates, especially given that some matrices, such as silica, may not be fully biodegradable. Long‐term studies in juvenile animal models must address the potential accumulation of nanoparticles in organs such as the liver, spleen, and brain, as well as the risks of chronic inflammation, glial activation, and oxidative stress [225, 226].

**Table 6:** Critical quality parameters of sol‐gel nanoparticles and their relevance to regulatory approval.

Critical quality parameters for sol‐gel nanoparticles	Relevance to regulatory approval
Drug loading and encapsulation efficiency	Determines therapeutic potential and consistency between batches
Particle size and morphology (via SEM/TEM, DLS)	Affects biodistribution and blood–brain barrier penetration
Surface area and porosity (BET analysis)	Influences release profile and degradation rate
In vitro drug release kinetics	Supports dose scheduling and pharmacodynamic modeling
Residual solvent content and endotoxin levels	Ensures biocompatibility and sterility
Stability under environmental and physiological conditions	Determines shelf life and field usability

To ensure safety and consistency in these systems, a robust manufacturing protocol must be established under GMP conditions. The sol‐gel process is inherently sensitive to changes in reaction parameters, including precursor concentration, solvent polarity, pH, catalyst type, and reaction temperature [227]. Slight variations can significantly impact particle size, porosity, and drug entrapment efficiency. Therefore, standard operating procedures (SOPs) and reproducible synthesis protocols must be implemented, with clear quality control checkpoints [228].

Different dosage forms of sol‐gel systems—such as injectable gels, monolithic implants, or nasal sprays—each require specific packaging, sterilization techniques, and stability assessments [229]. For example, injectable systems must demonstrate in situ gelation under physiological conditions, whereas nasal formulations must comply with standards for mucosal irritation and delivery efficiency [230, 231]. Sterilization must preserve both nanoparticle integrity and the bioactivity of the encapsulated drug. This requirement is often challenging for lipid‐based or polymeric systems but is more feasible with sol‐gel materials due to their structural stability [178].

Clinical trial design in pediatric epilepsy introduces further complexities. Phase I and II trials should focus on age‐specific safety, tolerability, and preliminary efficacy. Participant selection must reflect the diversity of epileptic syndromes, including LGS and Dravet syndrome, which often present with polytherapy resistance and comorbid developmental delay. The evaluation of seizure control should rely on a combination of EEG monitoring, seizure diaries, and clinical assessments. At the same time, pharmacokinetic measurements may require cerebrospinal fluid sampling or imaging‐based biodistribution tracking [232, 233].

Importantly, trials should also include endpoints that reflect broader quality‐of‐life outcomes, such as improvements in sleep, cognition, behavioral stability, and caregiver burden. Given the chronic nature of epilepsy, long‐term monitoring is essential to detect delayed toxicity, particularly regarding cognitive development and neuroplasticity in children. Special attention must be paid to developmental pharmacokinetics, as drug metabolism, brain permeability, and immune responses differ markedly between infants, children, and adolescents [232, 233].

Ethical considerations are also central to the translational process. In pediatric research, informed consent must be obtained from parents or legal guardians. However, assent should also be sought from children old enough to understand the purpose and risks of participation. Transparency regarding the experimental nature of nanotherapies is essential, and risk–benefit assessments should err on the side of caution in early‐phase trials. The nonreversibility of some nanoparticle implants and the limited understanding of long‐term CNS exposure further justify rigorous oversight [221, 234].

Finally, the issue of equitable access cannot be overlooked. Although sol‐gel systems may offer a pathway to reduce long‐term treatment costs through depot formulations and improved seizure control, without deliberate policy mechanisms, such technologies may remain inaccessible to the very populations that need them most. In low‐resource settings, such as many regions of Latin America, affordability and distributed infrastructure are critical factors. Integration into public health systems, supported by cooperative manufacturing and pricing strategies, will be essential to avoid exacerbating existing disparities in pediatric epilepsy care [223].

In summary, the path from sol‐gel nanoparticle synthesis to clinical implementation in CRE requires coordinated advances in regulatory frameworks, preclinical validation, manufacturing quality, and ethical trial design. Addressing these translational challenges will be essential not only for enabling approval but also for ensuring that such innovations achieve their intended social and clinical impact.

## 10. Future Perspectives and Research Gaps

Despite the encouraging advances in sol‐gel–derived nanoparticle systems for the treatment of CRE, several key scientific, clinical, and logistical challenges must be addressed before these technologies can be translated into widespread clinical use. This section outlines the principal research gaps that persist across the domains of formulation science, preclinical validation, clinical trial design, and implementation in resource‐constrained healthcare systems—particularly in Latin America, where the burden of RE is disproportionately high.

From a materials science perspective, one of the foremost challenges is achieving precise control over drug release kinetics in the context of biological complexity. Although sol‐gel systems are inherently tuneable—by manipulating synthesis parameters such as solvent polarity, pH, precursor concentration, and catalyst choice—their behavior in vivo remains challenging to predict. Drug release in the CNS is influenced not only by the internal structure of the nanoparticle matrix but also by external variables such as enzymatic activity, cerebrospinal fluid dynamics, immune responses, and cellular uptake mechanisms. Future research should prioritize the development of predictive models and real‐time tracking tools that integrate physicochemical properties with biological responses, enabling more accurate forecasting of therapeutic windows and adverse effects.

Another important area for development is the codelivery of multidrug regimens. Polytherapy is frequently necessary in patients with CRE, particularly those suffering from syndromes such as LGS or Dravet syndrome, which are poorly controlled by monotherapy. Although sol‐gel systems enable the encapsulation of multiple agents, there is a lack of studies systematically exploring drug–drug interactions within a shared matrix, particularly with respect to differential release rates, stability, and mutual degradation. Formulations that can release drugs in a temporally staged or stimuli‐responsive manner are especially needed to reflect the dynamic therapeutic demands of complex epilepsy syndromes.

The route of administration also remains a relatively underexplored variable in sol‐gel epilepsy research. Most current investigations focus on stereotactic implantation or intracerebral injection—methods that, although effective, are invasive and require neurosurgical infrastructure. To enhance accessibility, especially in low‐resource regions, future research should explore minimally invasive delivery systems, including intranasal, subcutaneous, and transmucosal approaches. Thermosensitive sol‐gel precursors that form in situ depots upon contact with physiological fluids may offer a practical solution for sustained CNS delivery without requiring surgical intervention. Additionally, pediatric‐appropriate devices and dosage forms must be prioritized to improve treatment adherence and minimize administration‐related stress.

The long‐term biocompatibility and biodegradation of sol‐gel matrices also warrant further investigation. Although materials such as silica and titania have generally shown favorable safety profiles in preclinical models, the cumulative effects of chronic exposure—especially during critical periods of neurodevelopment—remain poorly understood. Studies must assess not only acute toxicity but also the potential for inflammatory sequelae, neuronal remodeling, or interference with synaptic plasticity, which could have long‐term neurocognitive consequences. Furthermore, the kinetics of matrix degradation and its clearance from the CNS remain inadequately defined. It is essential to determine whether these materials persist in brain tissue, are metabolized, or are transported to other organs over time, particularly when repeated administration is required.

In the realm of clinical research, there is an urgent need for more comprehensive translational frameworks that bridge the gap between experimental nanomedicine and real‐world pediatric neurology. Immensely few sol‐gel formulations have advanced beyond in vitro or rodent models. Larger animal models, such as nonhuman primates or porcine systems, may provide more physiologically relevant data regarding drug distribution, metabolism, and immune response. Furthermore, there is a pressing need for early‐phase clinical trials that are ethically designed and methodologically robust, particularly in pediatric populations where trial enrollment, informed consent, and long‐term follow‐up are uniquely complex.

A persistent barrier to translation is the lack of standardization in nanoparticle characterization and reporting. Variations in synthesis techniques, measurement protocols, and outcome metrics make it difficult to compare results across studies or aggregate findings into meta‐analyses. International guidelines for nanoparticle reporting, including size distribution, zeta potential, encapsulation efficiency, and release kinetics, would greatly facilitate reproducibility and regulatory acceptance.

At a broader systems level, the integration of sol‐gel therapies into public health infrastructure remains a formidable challenge—particularly in Latin America, where disparities in epilepsy care are linked to socioeconomic, geographic, and cultural factors. Although sol‐gel systems hold promise for decentralizing treatment through long‐acting, implantable or injectable formulations, their success depends on coordinated investment in healthcare delivery mechanisms, including training of personnel, provision of diagnostic tools (e.g., EEG and MRI), and establishment of supply chains for nanomedicine components. Implementation science approaches may help map the contextual factors that affect adoption, including caregiver perceptions, clinical training, and logistical feasibility in low‐resource environments.

In addition, the economic evaluation of sol‐gel therapies is a critical but underdeveloped area. Initial production costs, particularly for GMP‐compliant synthesis, may be high; however, these must be weighed against potential cost savings from reduced hospitalization, fewer adverse effects, and improved seizure control. Cost‐effectiveness studies that incorporate health system modeling and quality‐adjusted life years (QALYs) will be essential to justify public investment and guide equitable pricing strategies.

Lastly, the future of sol‐gel applications in epilepsy may be significantly enhanced by the convergence of nanotechnology with personalized medicine. Advances in genomics, EEG‐based seizure mapping, and machine learning–based prediction models could enable the tailoring of nanoparticle compositions to individual patients based on genetic mutations, seizure patterns, or metabolic profiles. Research in this area remains nascent but highly promising, and interdisciplinary collaboration will be key to unlocking these capabilities.

In conclusion, although sol‐gel–based nanoparticles offer a powerful new paradigm for the treatment of CRE, substantial knowledge gaps and infrastructure must be addressed before this potential can be fully realized. Addressing these gaps through targeted research, clinical innovation, and policy reform will be critical to ensuring that nanomedicine becomes not only a scientific advance but also a tool for equity and impact in global pediatric neurology. Despite these promising advances, several barriers remain before clinical implementation becomes feasible. Standardization of nanoparticle synthesis, large‐scale GMP manufacturing, long‐term safety evaluation, pediatric toxicology, regulatory harmonization, and well‐designed clinical trials remain essential steps toward clinical translation.

## Author Contributions

All authors contributed equally to the writing of the manuscript. All the authors edited the manuscript.

## Funding

No funding was received for this manuscript.

## Disclosure

All authors approved the final version of the manuscript.

## Ethics Statement

The authors have nothing to report.

## Consent

The authors have nothing to report.

## Conflicts of Interest

The authors declare no conflicts of interest.

## Data Availability

The data that support the findings of this study are available from the corresponding author upon reasonable request.
